# Biomechanical and biological responses of periodontium in orthodontic tooth movement: up-date in a new decade

**DOI:** 10.1038/s41368-021-00125-5

**Published:** 2021-06-28

**Authors:** Yuan Li, Qi Zhan, Minyue Bao, Jianru Yi, Yu Li

**Affiliations:** State Key Laboratory of Oral Diseases & National Clinical Research Center for Oral Diseases & Department of Orthodontics, West China Hospital of Stomatology, Sichuan University, Chengdu, China

**Keywords:** Cell biology, Bone quality and biomechanics

## Abstract

Nowadays, orthodontic treatment has become increasingly popular. However, the biological mechanisms of orthodontic tooth movement (OTM) have not been fully elucidated. We were aiming to summarize the evidences regarding the mechanisms of OTM. Firstly, we introduced the research models as a basis for further discussion of mechanisms. Secondly, we proposed a new hypothesis regarding the primary roles of periodontal ligament cells (PDLCs) and osteocytes involved in OTM mechanisms and summarized the biomechanical and biological responses of the periodontium in OTM through four steps, basically in OTM temporal sequences, as follows: (1) Extracellular mechanobiology of periodontium: biological, mechanical, and material changes of acellular components in periodontium under orthodontic forces were introduced. (2) Cell strain: the sensing, transduction, and regulation of mechanical stimuli in PDLCs and osteocytes. (3) Cell activation and differentiation: the activation and differentiation mechanisms of osteoblast and osteoclast, the force-induced sterile inflammation, and the communication networks consisting of sensors and effectors. (4) Tissue remodeling: the remodeling of bone and periodontal ligament (PDL) in the compression side and tension side responding to mechanical stimuli and root resorption. Lastly, we talked about the clinical implications of the updated OTM mechanisms, regarding optimal orthodontic force (OOF), acceleration of OTM, and prevention of root resorption.

## Introduction

Orthodontic treatment is aiming to move malpositioned teeth to an appropriate position through the remodeling of the periodontium stimulated by orthodontic force. The underlying biomechanical and biological mechanisms of orthodontic tooth movement are essential for efficient and safe orthodontic treatment. Since the first publication regarding the mechanism of OTM in 1911, several theories had been proposed^1^. Up to now, the compression–tension theory is well accepted and proposes that cellular responses are modulated by chemical messengers, released from blood flow or cells in situ, in response to mechanical stress imposed on the periodontal ligament and alveolar bone. However, the detailed mechanisms of OTM still remain to be elucidated. In recent years, abundant new findings related to biomechanical and biological changes in periodontium during OTM have been published. In this study, we summarized the knowledge of OTM mechanisms mainly based on studies published in the past decade, and provided an up-date review for the new decade, with the focus on sequential biomechanical and biological responses of the periodontium in OTM, and their relevant clinical implications.

## Results

### Study selection

The initial literature search yielded 6 808 papers. 2 863 articles were selected after removing duplicates, in which, 1 946 studies were excluded for irrelevance. After reading the full text, 317 of the remaining 487 articles were excluded because of their low quality or lack of relevance. Ultimately, 170 studies were included in this review. The reasons for exclusion are noted in the Supplementary materials.

### Synthesis of results

According to the results of the entitled studies, 15 studies were related to the different types of research models for OTM researches and were discussed at first. Totally, 118 studies focused on the exact mechanisms of OTM, including the mechanobiology, cytobiology, and immunology of periodontium and osseous tissue, which were divided into four steps for clear logic. Finally, 37 studies reported the cutting edge developments of OOF, acceleration of OTM, and prevention of root resorption, and they were synthesized in a section for understanding clinical implications of OTM mechanisms.

## Discussions

### Research models

Generally, there are three categories of experimental models for investigating biomechanical and biological responses of the periodontium in OTM, including the in vivo, in vitro, and analytical models. Different models are used for research purposes, and an integrative study based on multiple experimental models can reach more comprehensive and reliable conclusions.

#### In vivo models

Animals including rats, mice, dogs, monkeys, and rabbits have been adopted for in vivo exploration of OTM mechanisms. Among them, rats, including Sprague Dawley and Wistar strains, are the most common experimental animals due to the low cost, short growth cycle, and similar molar structure with humans. Mice, mainly the c57BL/6J strain, are the second most used animal model^2^. In murine OTM models, the orthodontic force can be applied with a palatal expansion wire spring^3^, a NiTi coil spring to mesialize the molar, which is an optimal choice for OTM beyond 7 days^4,5^, or a separating rubber band, which is recommended only for short-term observation^6^. Recently, a rat OTM model was established which involved microscope camera and cone-beam computed tomography (CBCT) images for more precise analyses of differentiated OTM, so as to investigate the individual contribution of tooth tipping, body translation, and root torque to overall displacement^7^. Animal models are the earliest models established to study the orthodontic mechanisms, and the findings have essential implications for the clinical intervention of OTM, avoiding complications and controlling treatment duration. However, differences in dentoalveolar structure and force system between human and animal OTM models might affect the accurate extrapolation of the conclusions from animal to human. For instance, a narrative review suggested that the rodent model can only be used to understand the initial phases of OTM rather than the prolonged adaptation in response to bodily tooth movement^8^.

In summary, in vivo animal models are the most important and reliable tools for studying biological mechanisms in OTM, which call for further improvement to provide more precise orthodontic force and tooth movement.

#### In vitro models

In vitro models have been established to elucidate the mechanisms of how cells sense, respond to and transform specific mechanical stimulations to molecular signals. Studies on OTM mechanisms have focused on periodontal ligament fibroblasts (PDLFs)/PDLCs, periodontal ligament stem cells (PDLSCs), and osteoblasts (OBs). It is worth noting that PDLFs are the major components of PDLCs and we are going to call them PDLCs uniformly, for a more accurate definition. In most of these studies, PDLCs are two-dimensionally (2D) cultured, whilst novel three-dimensional (3D) culture and force loading models for PDLC have also been reported in the past decade^9^. In 3D culture models, the cells are grown in a geometrical constitution more similar within the vivo situation, therefore they may well respond to mechanical stimulation in a more realistic pattern. In contrast to traditional collagen gel, a thin sheet of porous polylactic-co-glycolic acid (PLGA) may better serve as a scaffold for the 3D culture of PDLC in the “periodontal tissue model”^10^, due to the porosity and elastic modulus more similar with human PDL. Notably, scaffold materials in such 3D culture models can affect the cellular mechanical responses, for instance, the gene expression profiles of PDLCs in PLGA are substantially different from that in the collagen gel^11^. Therefore, the optimal scaffold material and its conditions should be verified and standardized for specific cells and research purposes. The in vitro mechanical loading system should also be best designed to mimic the authentic in vivo force modality. For instance, the weight loading approach, generating static unidirectional compressive stress, is suitable to mimic orthodontic force, whilst the substrate deformation-based loading approach, generating cyclic tension, may better mimic the masticatory force^12^. In such in vitro models, primary human PDLCs have been most commonly used, which suffer from the need for human donors and limited proliferative capacity^9^. Recently, Weider et al.^13^ showed that an immortalized cell line, PDL-hTERT, derived from primary human PDLFs, exhibited characteristic responses to weight-mediated compressive force resembling those of primary cells. What’s more, in vitro 3D cementocyte differentiation scaffolds were also established for studying orthodontic-induced root resorption^14^.

In summary, the in vitro models are important for studying cytomechanics in OTM, and 3D culture models which better simulate the in vivo environment may be more promising in future research.

#### Analytical models

Finite element analysis models are models which introduce a finite number of elements and nodes to represent geometry. These models were primarily applied to dental research in 1973 for analysis of the stress and strain in the alveolar tissues^15^. Up to now, finite element analysis models have been adopted in multiple areas of dentistry, including OTM. For obtaining the scanning images of the research objects, the main tools used are magnetic resonance imaging, spiral CT, and CBCT, among which CBCT is the first choice. Then 3D reconstruction of the images is required, for which Mimics is the most preferred software. At present, the mainstream software of finite element analysis is Ansys and Abaqus. While the former possesses better ability in specific analysis, the latter is easier to operate and saves time^16^. Currently, finite element analysis models have been predominantly adopted to explore mechanisms of orthodontic appliances, properties of the wires, and mechanical analysis of orthodontic anchor screws. These models are characterized by high efficiency, accuracy, and low cost. However, since teeth and periodontal tissues are anisotropic and heterogeneous, different parts should be endowed with different material properties. In most existing researches, the materials involved were assumed to be homogeneous and isotropic linear elastic materials, which diverge from the fact. For example, a study firstly included the PDL fibers in a model with realistic tooth and bone geometry and the inclusion of PDL fibers alters the strains in the mandibular bone, increasing the strains in the tooth socket compared to PDL modeled without fibers^17^. Recently, a study showed that the locations of the center of resistance in the four model configurations comprising the combinations between linear or non-linear material models and uniform or realistic PDL thicknesses were in the range 37.2–45.3% (root apex: 100%; alveolar margin: 0%)^18^. Orthodontic load variation within the clinical practice range resulted in the center of resistance variations below 0.3%. Therefore, the simplest model configuration with linear material model and uniform PDL thickness appears sufficiently accurate for clinical practice.

In summary, the finite element analysis model is especially useful for studying periodontal material mechanics in OTM, with the need to further verify, simplify and unify relevant parameters.

### Hypothetical theories and four steps for mechanisms of OTM

**Fig. 1:**
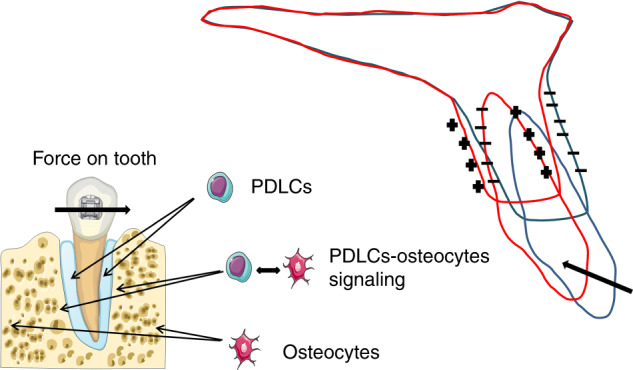
When a maxillary incisor is retracted, the bone resorption (−) occurs in the compression side and in the labial side of the alveolar bone, while bone deposition (+) in the tension side and in the palatal side of the alveolar bone. To explain the mechanism, we hypothesis that the PDLCs and osteocytes are the primary sensors responding to mechanical signals and the PDLCs control the soft tissue remodeling, while a PDLC-dependent PDLCs-osteocytes signaling network control the internal hard tissue remodeling and osteocytes control the external hard tissue remodeling

As is well-known about the mechanism of OTM, the compression–tension theory proposes that under the orthodontic force, periodontium can be divided into the compression side and the tension side around the stressed teeth, with osteoclastogenesis in the compression side and osteogenesis in the tension side. This theory emphasized the internal surfaces of alveolar bone but ignored the external surfaces. For example, when a maxillary incisor is retracted, we can see the bone resorption in the compression side and in the labial side of alveolar bone, while bone deposition in the tension side and in the palatal side of alveolar bone, which is displayed in Fig. 1. This phenomenon was previously explained by the “deflection” and “biologic-electricity” theory that the internal surface of the left cortex in Fig. 1 elongates in tension, while the external surface shortens in compression. Increasing concavity has consistently been shown to be associated with electronegativity and bone formation. In contrast, increasing convexity is associated with electropositivity and bone resorption^19^. In the 1960s, electrical potentials are believed responsible for regulating bone formation and bone resorption, before many biochemical mediators were found. As the opinion of Meikle^19^, stress-generated electrical potentials only represent a by-product of deformation and a physical phenomenon. Recently, another theory called the Biphasic Theory divides OTM into the initial Catabolic Phase, during which osteoclasts (OC) resorb bone at both compression and tension sites, and the Anabolic Phase, which occurs subsequently to restore the alveolar bone to its pretreatment levels. The Biphasic Theory affirmed the fact that the PDL was the primary target of orthodontic force and induced inflammation-dependent osteoclastogenesis for the Catabolic Phase. The Anabolic Phase is mainly based on the fact that osteoblast activation requires intermittent loads of specific frequency and acceleration at physiologic levels in long bones and alveolar bone, while the orthodontic tensile force, which is a static force, causes bone resorption on long bones^20^. This theory provoked the thinking about the types of force and relative cell responses. To explain the inconsistent phenomenon with OTM that the bone under loading is osteogenic and under release it is resorptive, there were primarily two explanations described in Wise’s review^21^. It is obvious that compression force superimposes the tissue injury onto the physiological response, which produces resorptive inflammatory products to absorb the injured tissue. It is proved that orthodontic force induces systemic immune responses within periodontal tissues associate with the recruitment of the systemic inflammatory monocytes and multiple inflammatory factors^22^. This may be the reason why the existence of PDL causes totally adverse bone remodeling effects under mechanical force. Another theory proposed that osteoclastic activities at compression sites can be considered as a consequence of loss of the functional strain from the PDL, while osteogenic activities at tension sites can be a result of loading of the PDL fibers. Therefore, the key of this question can be attributed to the existence of the PDL, which senses the mechanical stimuli primarily and initiate downstream signal responses including cell activities and inflammation.

**Fig. 2:**
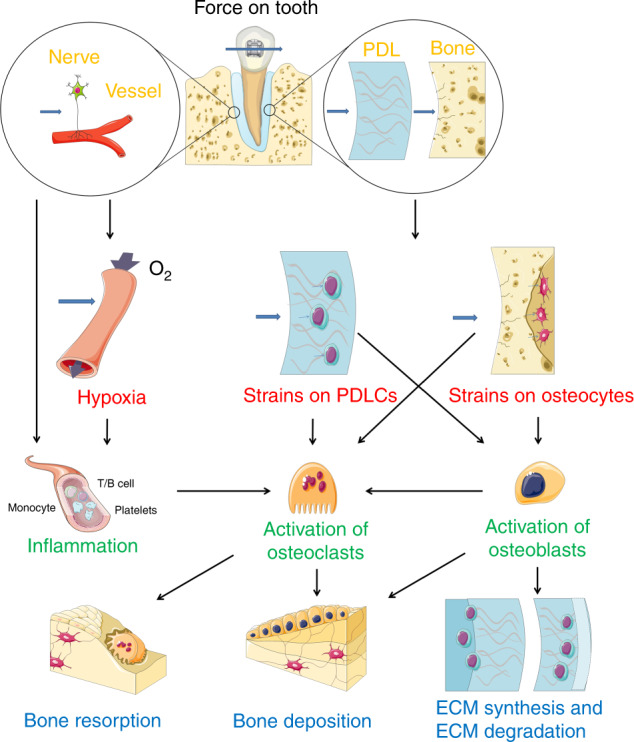
The process of the transduction from mechanical loadings to biological signals. Step 1: the extracellular mechanobiology of the periodontium (in yellow). Step 2: cell strains (in red). Step 3: cells activation and differentiation (in green). Step 4: tissue remodeling (in blue)

Based on the progress in the OTM mechanisms, we proposed a new hypothetical theory that during OTM, the PDLCs and osteocytes are the primary sensors responding to mechanical signals and the PDLCs control the soft tissue remodeling, while a PDLC-dependent PDLCs-osteocytes signaling network controls the internal hard tissue remodeling and osteocytes control the external hard tissue remodeling (Fig. 1). Previous critical reviews, such as V. Krishnan’ published early in 2009^23^, has divided the process of the transduction from mechanical loadings to biological signals into four steps: (1) matrix strain and fluid flow, which is basically regarding the extracellular mechanobiology of the periodontium, (2) cell strain, (3) cell activation and differentiation, and 4) tissue remodeling (Fig. 2). In the first step, we mainly introduced the extracellular matrix (ECM) changes in responding to force in PDL and alveolar bone, mainly including matrix deformation and the subsequent fluid flow alteration. The neurovascular system responses were also involved. We emphasized the important role of the PDL and its material properties. In the second step, the mechanical signals were transduced through ECM to the mechanosensory cells (PDLCs and osteocytes) and activated intracellular signaling pathways, leading to primary cell responses. Some new mechanisms including non-coding RNAs, hypoxia, and autophagy were developed in recent years. In the third step, we summarized the regulatory mechanisms of activation and differentiation of OBs and OCs as the basis of PDLCs- and osteocyte-regulated downstream mechanisms. A network including PDLCs–OBs/OCs, osteocytes–OBs/OCs, and PDLCs–osteocytes signaling are the key for our hypothesis. In the fourth step, responsive matrix enzymes are secreted, leading to tissue remodeling including synthesis or degradation in PDL, as well as deposition or resorption of alveolar bone. The root resorption was also briefly described due to its inevitable damage during OTM.

### Step 1: extracellular mechanobiology of the periodontium

#### Matrix strain and fluid flow

On the histological level, once a force is applied and the tooth moves, relative to the fixed socket, the tooth along with its adjacent periodontium will be compressed on one side and stretched on the other side. On the compression side, the force leads to compression of PDL fibers and subsequently the alveolar bone. On the tension side, the force leads to the stretching of PDL fibers and the alveolar bone. Both strains cause matrix deformation and fluid flow alteration in turn. In the alveolar bone, according to the matrix deformation hypotheses, the application of macroscopical compressive force to bone leads to a magnified local microscopic strain, inducing bone matrix deformation, damages, and microcracks. The accumulation of microcracks represent the first damage caused by mechanical loads and result in additional cellular damage to osteocytes in the microcrack regions, thereby inducing osteocyte apoptosis, osteoclastogenesis and bone resorption^24^. However, high-magnitude forces still cannot move an implant, which indicates that the microcrack is not the essential mechanism triggering OTM. According to the fluid flow hypotheses, in the alveolar bone, force is converted to immediate fluid flow alteration in the lacunar–canalicular system, followed by increasing shear stress on osteocytes^25^. It is suspicious that the immediate shear stress can cause prolonged cell responses and tissue reactions. In the PDL, matrix strain causes the compression or stretching of collagen fibers and induces configurational changes of the ECM proteins, while the fluid is squeezed or inhaled, inducing the cell strains in PDLCs. These mechanical stimuli both in PDL activate mechanosensory cells, activate intracellular signal transductions, and finally induce differential cell responses.

#### Neurovascular system

In addition to the matrix, the neurovascular system also plays an important role in bone pathology. The nerve endings within paradental tissues mainly consist of mechanoreceptors and nociceptors. The use of beta-adrenergic receptor blockers such as atenolol decreased tooth movement, suggesting the regulation of bone metabolism by the sympathetic nervous system^26^. The sympathetic nervous system regulates bone resorption mainly through the β-2 adrenergic receptor (Adrb2), one of the three postsynaptic β adrenergic receptors. Adrb2^−^^/^^−^ mice showed a higher bone mass phenotype by decreasing the expression of Receptor Activator of Nuclear Factor-κ B Ligand (RANKL) in OBs and blockage of sympathetic nervous activity by superior cervical ganglion ectomy in the jawbones of rats reduced OTM distance, while the injection of nonselective Adrb2 agonist accelerated OTM. Mechanistically, compression enhanced PDLCs’ Adrb2 expression through the elevation concentration of intracellular Ca^2+^, finally increasing the RANKL/osteoprotegerin (OPG) ratio and OC differentiation^27^. When nerve endings are distorted upon application of orthodontic forces, they release vasoactive neuropeptides such as a vasoactive intestinal polypeptide, calcitonin gene-related peptide, and substance P, which interact with vascular endothelial cells (ECs), stimulating plasma extravasation and migration of circulating leukocytes, monocytes, and macrophages by adhesion to receptors on these cells, and finally providing sufficient support for OC differentiation^23^.

Blood vessels in the PDL provide nutrients, immune cells, and hormones required for orthodontic-induced remodeling of their strained surrounding tissues. The ECM surrounding the vessels provides critical support for the vascular endothelium through the adhesion of the ECs to the ECM. The vascular endothelial growth factor (VEGF) locate in ECs, mononuclear cells, OBs, OCs, fibroblasts, and local necrotic areas in the compression zone and locate in fibroblasts and OBs in the tensile zone during OTM^28^. Wu et al.^29^ found that coculture of PDLSCs and ECs increase the expression and release of VEGF under the hypoxia compared with PDLSCs cultured alone, suggesting that PDLSCs or ECs may interact with each other by producing VEGF. Under the hypoxia, coculture of PDLSCs and ECs significantly activates the MEK/ERK and p38 MAPK signal cascades to stimulate the expression of Runt-related transcription factor 2 (Runx2) and promote osteogenic differentiation^30^. Collectively, nerves and blood vessels take part in the OTM and regulate osteogenesis or osteoclastogenesis both through indirect and direct ways, although the mechanisms are not clear enough.

#### Dentoalveolar fibrous joint

For another perspective, the dentoalveolar fibrous joint was proposed in Lin’s previous review^31^. The interfaces within the joint, including the interface of PDL and bone and the interface of PDL and cementum, were emphasized as the determining factor of the mechanical properties, rather than one part of the joint. An interface is a functionally graded transition of dissimilar materials from softer to harder tissues and a gradient in mechanical stiffness caused by a gradual change in inorganic to organic ratio, which has developed to reinforce underlying tissue structure and resist the external strains. In addition, hyperfunction induced by external loadings upregulates osteoclastic response to increasing the general 150–380 μm-wide PDL space as a response and cushion, while hypofunction decreases the PDL width. Any region that narrows than the PDL space requires tissue resorption not limited to the bone but also occurred in cementum, with aim of maintaining a functionally viable PDL–space for tooth^32^. Definitely, this resorptive process at the PDL–bone interface can occur at a significantly higher rate than that at PDL–cementum interface. Elucidating the localization and function of biomolecules specifically at interfaces is critical for OTM development and control. And the “dentoalveolar fibrous joint” theory provides new insight into the tooth movement principle.

#### Substrate rigidity of PDL

Since the teeth–alveolar bone interface is a gradient in mechanical stiffness, the force-induced changes in substrate rigidity may be an important factor in maintaining the PDL width and regulating tissue remodeling. Westover et al. used the Advanced System for Implant Stability Testing system to measure the PDL stiffness change of human maxillary canines during OTM and showed an average maximum reduction in stiffness of 73.4% ± 7.7% of all patients^33^. However, neither the relationship between PDL stiffness and OTM result nor the stiffness changes in compression and tension side were provided. Therefore, we can only hypothesize that substrate rigidity can influence the cell activities during OTM, which was rarely reported in the past. We provided some evidence in other fields and they should be proved during OTM.

Initially, substrate rigidity affects the cell adhesion, which can be reflected by the focal adhesions and cytoskeleton proteins^34^. Early studies have found that fibroblasts surrounded by stiff and highly cross-linked gels presented stable focal adhesions, while it presented diffuse and weak adhesions surrounded by soft and lightly cross-linked gels^35^. Furthermore, substrate rigidity also affects the maturation process of focal adhesions. Spreading fibroblasts exhibit stereotypic, spatially isotropic on the solid substrates, and the focal adhesions experience a reproducible sequence of functional stages from initial contacts to maturation, while on the soft surface, only the initial contacts of fibroblasts can be detected^36^. Jalali et al. directly quantified the reality that maximum detachment force of endothelial cells increased with increasing substrate rigidity^37^. In general, substrate rigidity influences the adhesive strength of cells by altering the properties of focal adhesions and cytoskeleton proteins. However, focal adhesions do not continue to aggrandize when the substrate becomes too stiff. Due to the high substrate rigidity, the amount of integrin is saturated, and the adhesive force cannot be redistributed, which leads to the disintegration of adhesive spots^38^.

Besides, substrate rigidity exerts long-term effects on cell differentiation and it has been acknowledged that substrate rigidity has links with the direction of cell differentiation. It was reported that hard substrate could give rise to differentiation of mesenchymal stem cells (MSCs) and PDLSCs into OB, while soft substrate promotes their differentiation into adipocytes^39^. However, current studies have not reached a consensus on how the rigidity of different substrates affects cell differentiation. Some findings suggested that differentiation of stem cells was affected by collagen crosslink concentration but not elastic modulus of the substrates^40^. Furthermore, surface proteins also determine the direction of cell differentiation. According to Choi et al.^41^, laminin enhanced stem cell differentiation into adipocytes, while fibronectin promoted differentiation into OB. The mechanism was focused on the link between nuclear proteins and membrane proteins via the cytoskeleton. The phenomenon was observed in various cell systems including bone marrow-derived MSCs, which resembled PDLSCs in multiple aspects, thus might provide us new insight for OTM mechanisms^42^.

In conclusion, the substrate rigidity of PDL plays an important role in cell activities via its influence on the configurational changes of ECM, cytomembrane, and cytoplasmic proteins. It is a pity that we have not found enough relevant studies investigating the substrate rigidity changes during OTM and its effects on PDLCs. Therefore, the substrate rigidity of PDL is a potential direction of OTM-related researches to further supplement the regulatory mechanism interpretation of the matrix microenvironment under orthodontic force application.

### Step 2: cell strain

We believe that the fibroblasts in the PDL and the osteocytes in the alveolar bone are primary mechanical sensors that initially accept the external mechanical signals and are responsible for the main regulation of tissue remodeling. Therefore, the main object of the second step is to answer the question how these sensors translate the mechanical signals to biological signals. Based on a large number of manuscripts, we summarized the receptors, channels, cellular signal pathways, and intranuclear signal pathways in PDLCs and osteocytes, which provide abundant biologic bases for experimental or clinical intervention. In addition, some new mechanisms are proposed in recent years, including the myofibroblast, noncoding RNAs, hypoxia, and autophagy. These new ways expand our horizon about other strain-induced regulators and strain-cooperated regulators.

#### Mechanical signal transduction in PDLCs

PDLCs may be the prior cellular receptor in PDL response to mechanical signals because we have saw the induction of genes involved in bone remodeling, inflammation, ECM reorganization, and angiogenesis in human PDLCs stimulated with physiological orthodontic compressive forces within 24 h^43^. The connection of matrix strain with cell strain is through the focal adhesion domains (FADs) located at the cell membrane, which comprise transmembrane integrins and cytoplasmic focal adhesion proteins, including focal adhesion kinase (FAK), Src, paxillin, tensin, and filamin. They physically bind to ECM fibronectin, vitronectin, and collagen extracellularly, and cytoskeleton proteins such as talin, vinculin, α-actinin, and paxillin intracellularly. The FADs are responsible for cell adhesion, receiving force stimuli and activating intracellular molecules. Integrins are heterodimeric receptors made up of structurally distinct α and β subunits, binding to ECM proteins via the RGD (arginine–glycine–aspartate) peptide sequence^44^. Different binding properties of integrins depend on its different conformational changes, for instance, α2β1 integrin binds to collagen and laminin while αvβ3 integrin binds to vitronectin. The cytoskeleton consists of three main polymeric elements including actin filaments, microtubules, and intermediate filaments, providing resistance to strain-induced deformation. Cytoskeleton contacts with the nucleus mainly through the outer nuclear membrane proteins nesprins and inner nuclear membrane proteins SUN1, SUN2, and Lamins, mediating transmission of mechanical load to the nuclear DNAs and proteins^45^. The ECM proteins, FADs, cytoskeleton, and nuclear proteins ultimately constitute a molecular link for mechanical signal transduction, initiated by the configurational integrin changes resulted from the stretch of the ECM proteins. For example, the activation of integrin αvβ3 stimulated by mechanical stretch induces an increasing integrin-binding force to ECM proteins and starts the downstream signal responses. The intracellular signal pathways activated by integrins configurational changes mainly include the guanosine triphosphatases (GTPases). Rho family GTPases such as Rho, Rac, and Cdc42 are involved in focal adhesion proteins and actin assembly, stress fiber formation, and formation of filopodia in fibroblasts via the Rho- mDia1 pathway^46^. mDia1, a member of the formin homology family of proteins, has the ability to regulate cellular cytoskeleton remodeling processes such as cytokinesis, polarity, motility, stress fiber formation, and neurite outgrowth. mDia1 functions as the effector of a small Rho GTPase that regulates actin cytoskeleton remodeling through its interaction with the actin polymerizing protein profilin-1. Profilin-1 has been considered a significant factor in the actin cytoskeleton, which is assumed to modulate actin dynamics by promoting both actin filament and depolymerization. Rho GDP dissociation inhibitor (RhoGDI) is the primary regulator of the activation of Rho GTPases, which can regulate the transformation between Rho-GDP and Rho-GTP. Notably, the hPDLCs cultured under cyclic strain showed increased expression levels of RhoA-GTP, profilin-1 protein, and the combination of RhoA and mDia1, whereas the expression levels of Rho-GDI were reduced. Furthermore, the cytoskeletal rearrangement of cells was enhanced. Profilin-1 protein expression and cytoskeletal reorganization under cyclic strain decreased due to the mDia1-siRNA transfection and the RhoGDI, whereas Rho-GDI siRNA transfection had the opposite effect on hPDLCs. Thus, the above experimental results demonstrated that Rho-GDI was an important protein in cyclic-strain-induced hPDLCs’ cytoskeletal rearrangement through regulating RhoA-GTP and mDia1^47^.

**Fig. 3:**
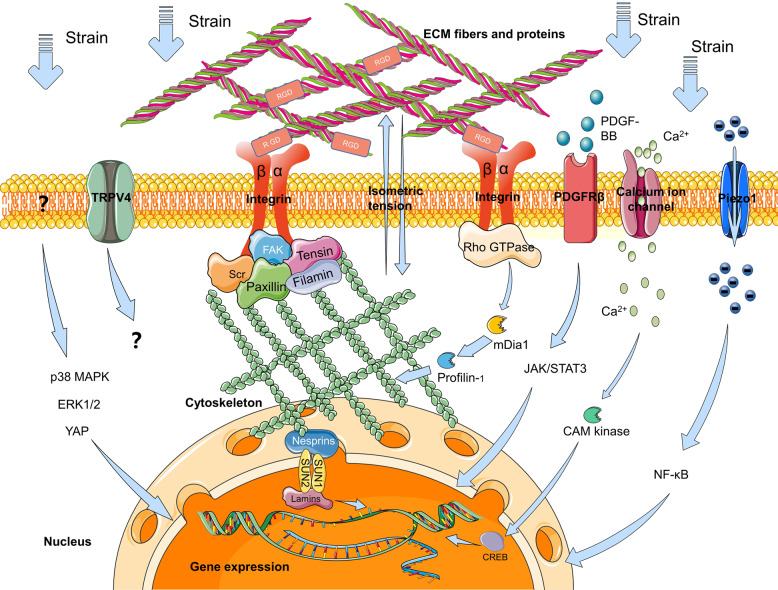
Hypothetical mechanisms of mechanical signal transduction in PDLCs

Besides the integrin, other mechanosensitive membrane receptors such as PDGFRβ and mechanosensitive ion channels including calcium ions channel, the Piezo1 ion channel, and the transient receptor potential cation channel subfamily V member 4 (TRPV4) ion channel, were also found in PDLCs. Platelet-derived growth factor-BB (PDGF-BB) has been recognized as a key regulator in bone regeneration and binds to the extracellular domains of PDGF receptor PDGFRβ. In a rat OTM model, PDGF-BB level was remarkably enhanced at the tension side during OTM in parallel with the up-regulated PDGFRβ+PDLCs. In orthodontic force-treated primary PDLCs, PDGFRβ expression was confirmed to be increased and the PDGF-BB/PDGFRβ signals were relevant to the activation of JAK/STAT3 signals. The protein expressions of JAK2 and STAT3 were elevated in the PDL on the tension side. In vivo treatment of the inhibitors for PDGFRβ and JAK–STAT signals were capable of attenuating the osteogenic differentiation during OTM^48^. Thus, it can be concluded that tensile force-induced PDGF-BB activated JAK2/STAT3 signals and osteogenesis during OTM via the membrane receptor PDGFRβ. The calcium ion channel is considered to participate in mechanotransduction because compressive forces were observed to activate autonomous calcium ions responsive behavior with an increased percentage of responsive PDLCs both in vitro and ex vivo^49^. Responding to stretch, the influx of calcium ions induced the calcium/calmodulin-dependent protein kinase (CAM kinase) to phosphorylate transcription factors such as cAMP response element-binding protein (CREB). The expression of CREB at the tension site in the mouse OTM model and in the cyclic tension strain treated hPDLCs were upregulated, which promoted secretion of osteoblastic factors^50^. Piezo1, a novel evolutionarily conserved mechanically activated current channel, was currently found significantly upregulated under static compressive stimuli in isolated hPDLCs, along with the enhancement of NF-κB signaling pathway and osteoclastogenesis. The Piezo1 inhibitor repressed osteoclastogenesis in the mechanical stress-pretreated PDLCs^51^. In another study, the expressions of Piezo1 and TRPV4 in the hPDLCs were significantly increased at 8 h after loading, accompanied by increased expressions of monocyte-colony stimulating factor (M-CSF) and RANKL. Key PDLC biomarkers were suppressed after mechanical loading following treatment with the inhibitors of Piezo1 and TRPV4^52^. The roles and underlying mechanisms of Piezo1 and TRPV4 in PDLC during mechanical signal transmission need to be verified. In addition, some other potential pathways were also detected. For example, the expression level of p38 MAPK protein was increased with time prolonged both in PDL tissues of orthodontic patients and loaded PDLCs^53^. A static equiaxial strain also activated the expression of ERK1/2 and the Hippo pathway effector Yes-associated protein (YAP) in strained hPDLCs^54^. The relationship between potential receptors, ion channels and signal pathways mentioned above may be valuable objects in further studies, and they are summarized in Fig. 3.

It is also suggested that a tension-dependent cellular integrity known as “tensegrity” is physiological pre-existing in the cell and directly causes chromatin deformation, consequent regulation of transcription and protein production^55^. In the absence of the tensegrity, cells often experience apoptosis and are incapable to sense an external loading, while cells are more sensitive to mechanical loading when the magnitudes of external and internal strains are similar^56^. The reason is that cell strains will cause isometric strain intracellularly, which further induces configurational changes of ECM proteins, generating a positive feedback loop^23^. Intracellular strain is able to promote assembly of focal adhesion proteins and clustering of integrins, induce configurational changes of some cytoskeletal proteins, and finally control gene expression by influencing intracellular signaling pathways. Therefore, external force and internal actin cytoskeletal contractile force are both required for efficient transduction from mechanical to biological signals.

#### Myofibroblast

In recent years, myofibroblasts were found to be differentiated from fibroblasts and exist in the PDL both in vivo and in vitro under tensile loadings, along with the upregulation of the alpha-smooth muscle actin (α-SMA)^57^. α-SMA is a mechanosensitive protein located in the stress fibers. The synergic effect of stress fibers and α-SMA provide the contraction ability of myofibroblasts, which results in tissue contraction. Tenascin-C is another typical protein that functions antagonistically to disassemble FADs in order to avoid overstretching of cells. PDL myofibroblasts were also reported to produce collagen and osteocalcin positively, suggesting that myofibroblasts had the ability to take part in mechanical signal transmission and periodontal tissue remodeling^58^. The Wnt/β-catenin pathway and transforming growth factor-beta (TGF-β) were recognized as the prerequisites of myofibroblast differentiation. Under orthodontic load, increasing expressions of Wnt3α, TGF-β1, α-SMA, and tenascin-C in both tension and compression PDL regions were found, along with the stimulation of myofibroblast differentiation by TGF-β1^59^. YAP is a mechanical sensor and a cytoskeletal signal mediator in the nuclear, whose target gene is TGF-β1. The further study found that extracellular mechanical loadings induced the cytoplasmic RhoA/ROCK pathway and the intranuclear YAP accumulation, to activate TGF-β1 and RUNX2 transcription, followed by the differentiation from PDLCs into myofibroblast^60^. In conclusion, myofibroblast is a newly discovered cell taking part in OTM and it was potential to be one of the targets in regulating mechanical signal transduction and tissue remodeling. Its differentiation sources and process, signal transduction mechanisms and functional differences, and commonalities with other PDLCs are worth investigating.

#### Mechanical signal transduction in osteocytes

Osteocytes were traditionally considered to be inactive bone matrix placeholder cells and their mechanosensory properties which regulate OBs and OCs functions are taken into account nowadays. The ablation of osteocytes in vivo reduced the number of OCs at the compression site during OTM^61^. Osteocyte processes, integrins, and ion channels are considered to be involved in the mechanotransduction. Over osteocyte processes, highly heterogeneous fluid flow patterns produced by the roughness of canalicular walls caused cytoskeletal deformation and consequently amplified mechanical signals^62^. Reduction of the number of osteocyte processes under fluid flow shear stress reduced osteogenesis activities and increased osteoclastogenesis activities^63^. Integrin αvβ3 works as the receptor for the vitronectin, osteopontin (OPN), and ECM proteins in bone^64^. It may play a similar role in osteocytes as it does in PDLCs. Meanwhile, the intercellular communication mediated by gap junction is thought to participate in the transduction of cellular signals. The gap junction is a channel connecting the cytoplasm and passage communication of ions, metabolites, ATP, and Ca^2+^ of two adjacent osteocytes. The channel is comprised of two hemichannels, each of which belongs to one of the adjacent cells and is an assembly of 6 connexin proteins. The component of gap junctions and hemichannels, connexin 43, was upregulated in mechanical loading osteocytes^65^. Some other hemichannels located on the cell body membrane rather than processes membrane are in communication with the extracellular microenvironment via a distinct mechanism from the gap junction^66^. The importance of osteocyte in mechanical signal transduction is more and more apparent at present, and more studies should focus on the role of osteocyte during OTM.

#### Non-coding RNAs

With the development of cognitive level and detective technology in cellular regulation, not only the intercellular signal proteins but also some non-coding RNAs play important roles in regulating gene expression and translation, including circular RNAs (circRs), microRNAs (miRs), and long non-coding RNAs (lncRs), which were previously undetected mechanisms that modulated the OTM process.

MiRs could be mechanosensitive and emerge as critical post-transcriptional regulators in the bone-remodeling process. MiR-21 has been previously reported to mediate stretch-induced osteogenic differentiation of PDLSCs and support OCs differentiation in vitro. A study generated miR-21-deficient mice and showed that miR-21 responded to orthodontic force in periodontal tissue in a dose- and time-dependent manner and regulated the osteogenesis following OTM by regulating programmed cell death 4 (Pdcd4)^67^. MiR-21 and Periodontal ligament-associated protein-1 (PLAP-1), a newly discovered ECM protein in PDL were also involved in the reconstruction of periodontal tissue under the orthodontic force in rats, which may be another target^68^. Hypoxia-inducible factor-1 (HIF-1) also exhibited similar expression patterns with miR-21 in rat OTM models and human PDLCs exposed to a hypoxic environment. Furthermore, miR-21 increased HIF-1 expression and promoted osteogenic differentiation while miR-21 inhibitors suppressed HIF-1 expression and down-regulated the osteogenic markers^69^. In conclusion, the results revealed that miR-21 played important roles in osteogenic differentiation during OTM. In addition, mechanical force induced different expression levels of miR-34a in vivo and miR-34a improved proliferation and osteogenic differentiation of PDLCs under mechanical tension and compression in vitro^70^. Down-regulated miR-34 expression was positively correlated with matrix metalloproteases (MMP-2, MMP-9, and MMP-14) expression. The miR-34a transfection into hPDLCs inhibited the expression of MMPs, suggesting that miR-34a is also involved in the expression of MMPs during OTM^71^.

The miR microarray assay was used to screen for mechanosensitive miR changes during compression- or tension-induced PDLCs, identifying that miR-572,-663,-575,-3679-5p, UL70-3p, and-3198 were upregulated only by compression. Real-time RT-PCR confirmed that compression induced miR-3198 expression, but tension reduced it in human PDLCs^72^. An expression profiling study also found that miR-195-5p, miR-424-5p, miR-1297, miR-3607-5p, miR-145-5p, miR-4328, and miR-224-5p were core miRs to support osteogenesis in PDLCs induced by tension, while another found 9 osteogenesis-related miRs in stretched PDLCs including miR-221-3p, miR-138-5p, miR-132-3p, miR-218-5p, miR-133a-3p, miR-145-3p, miR-143-5p, miR-486-3p, and miR-21-3p, which were validated by RT-qPCR^73,74^. These findings may provide a reliable reference for future studies to elucidate the biological mechanisms of miRs. Moreover, the delivery methods for miRs treatment also need exploration. Yu et al. reported the in vitro and in vivo effects of miR-34a on enhancing osteogenic differentiation under orthodontic force using an N-acetyl-L-leucine-modified polyethyleneimine carrier^75^. MiR-29 also showed increased expression during six weeks of OTM in humans and exosomes in gingival crevicular fluid (GCF)^76^, indicating a potential regulatory role of miR-29 and a potential exosome-carried miR therapeutic system.

LncRs are gene expression regulators that have longer than 200 nucleotides to modulate target genes at the level of posttranscriptional repression and competitively bind to specific miR sites to regulate their expression levels. LncR Nron (long noncoding RNA repressor of the nuclear factor of activated T cells) was recently found highly expressed in OC precursors but downregulated during osteoclastogenesis. To find whether the lncRs could be potential targets to regulate osteoclastogenesis in future clinics, Zhang et al. generated osteoclastic Nron transgenic and osteoclastic knockout mouse models. Overexpression of Nron reduced the OTM rate and decreased the number of OCs by regulating nuclear factor of activated T-cells cytoplasmic 1 (NFATc1) nuclear translocation, while specific deletion of Nron in osteoclasts increased the OTM rate^77^. LncR DANCR has been proven to be involved in osteoblast differentiation. The expression of DANCR and Jagged1 protein was increased in the rat OTM model and human PDLCs treated with compression. Knockdown of DANCR could inhibit osteoclastogenesis and bone resorption in vitro and in vivo, while overexpression of Jagged1 reversed si-DANCR effect. Taken together, DANCR regulated OC formation via Jagged1^78^. Another lncR TUG1 was indicated to positively regulate OC differentiation by targeting v-maf musculoaponeurotic fibrosarcoma oncogene homolog B (MafB) in the CD14+ peripheral blood mononuclear cells, which needs confirmation in OTM models in the future studies^79^.

An expression profiling study explored the lncR landscape of PDLSCs subjected to compressive force and 90 lncRs and 519 mRNAs were differentially expressed, which were involved in the ECM organization and the cellular response to hypoxia, including eight lncRs of interest (FER1L4, HIF1A-AS2, MIAT, NEAT1, ADAMTS9-AS2, LUCAT1, MIR31HG, and DHFRP1)^80^. However, as the most abundant noncoding RNAs in vivo, the potential regulatory role of lncRs in bone remodeling urgently needs to be clarified. Another expression profiling study obtained IncR-mRNA intersections including 263 lncRs, 1 599 mRNAs, and 3 762 interacting pairs. Among them, DNAJC3-AS1, WDFY3-AS2, LINC00482, and DLEU2 in the pathways of PI3K-Akt signaling and focal adhesion might play crucial roles in orthodontic forces pathogenesis^81^. Making still further progress, based on the previous miRNA microarray analysis, a total of 1,339 and 1,426 differentially expressed lncRs and mRNAs were identified in hPDLSCs under force and the potential interaction networks of lncRs-miRs-mRNAs were constructed. It was found that lncRs and mRNAs could competitively interact with the same miR.

CircRs also play critical roles in signal transduction during cell proliferation, differentiation, and apoptosis in a post-transcriptional manner. Recently, Wang, H. investigated the circRs expression patterns in PDLSCs induced by mechanical force and found one circR may regulate the same or different miRs and one miR may interact with single or multiple circRs. For example, circR-3140 was highly and widely associated with miR-21, indicating the potential importance of the CircR-miR network^82^. Therefore, we summarize that the non-coding RNA networks are involved in the mRNA regulation of PDLSCs and seem to be closely related with the cell differentiation, ECM remodeling, and cell responses to hypoxia, which might provide a novel mechanism in the regulation of the clinical OTM process, and the whole network patterns are waiting for development.

#### Hypoxia

Compared to direct compression strain, hypoxia is a slower and longer-lasting method to promote OC formation. HIF-1 is a heterodimeric transcription factor composed of α (inducible) and β (ubiquitous) subunits activated by hypoxic conditions (0–2% O_2_), promoting angiogenesis, stimulating cell proliferation and preventing cell death. Since HIF-1α was discovered, many studies have explored its regulatory mechanism. Under hypoxic conditions, HIF-1α stably translocates to the nucleus, and HIF-1α binds to its dimeric partner HIF-1β in the nucleus to stimulate the expression of its target genes, such as RANKL in PDLCs, which contributes to increased osteoclastogenesis. In cells under chronic or extreme hypoxia, the protective function of HIF-1ceases and leads to cell apoptosis. HIF-1α also induced hypoxic apoptosis of osteocytes via the JNK/caspase-3 pathway and the apoptotic-osteocyte-mediated osteoclastogenesis in vitro^83^. HIF1A antisense RNA 1 (HIF1A-AS1) and HIF1A antisense RNA 2 (HIF1A-AS2) are two lncRNAs associated with HIF-1α mRNA. HIF1A-AS1originates from the 5′ end of the hif-1α gene while HIF1A-AS2 originates from the 3′ end and is complementary on the 3′ untranslated region (UTR) of HIF-1α mRNA. Chen et al. found that HIF1A-AS1 and HIF1A-AS2 were expressed in PDLCs for the first time, but only HIF1A-AS2 suppressed HIF-1α expression^84^. It was hypothesized that HIF1A-AS2 hybridized to the 3′ UTR to inhibit the HIF-1α mRNA translation and the osteogenic differentiation in PDLCs. Since HIF1A-AS1 locates at the nuclear membrane, but HIF1A-AS2 only accumulates in the nucleus, HIF1A-AS1 and HIF1A-AS2 are possible to have different responses to different stimuli, which might be related to the mRNA transduction from the nucleus into the cytoplasm^85^. Therefore, HIF1A-AS1 and HIF1A-AS2a may be involved in different regulatory mechanisms associated with HIF-1α mRNA.

As important factors having the ability to initiate tissue remodeling, the relationship and importance of cell strains and hypoxia are controversial. Previously, to investigate the isolated and combined effects of compression and hypoxia on the osteoclastogenesis of PDLCs, our group found that either compression or hypoxia alone significantly up-regulated the gene expression of pro-osteoclastic cytokines in the PDLCs and the combination of the two had significantly stronger effects than either stimulation alone. In addition, comparing the two stimulants, we found that the osteoclastogenic property of the PDLCs peaked earlier (at 6 h) in the compression group than in the hypoxia group (at 24 h)^86^. However, Ullrich recently seeded human primary PDLCs randomly in conventional plates with O_2_-impermeable membranes and in special plates with gas-permeable membranes, enabling the experimental separation of mechanical and hypoxic effects that occur concomitantly during OTM. The expression of HIF-1α, osteoblastic and osteoclastic markers during PDLC-mediated osteoclastogenesis were significantly elevated by mechanical loading irrespective of the oxygen supply, whereas hypoxic conditions had no significant additional effects. In the context of OTM, the hypoxic marker HIF-1α does not appear to be primarily stabilized by a reduced O_2_ supply but is rather stabilized mechanically, while HIF-1α stabilization in macrophages is rather induced via the decreased oxygen supply than via mechanotransduction^87,88^. Different conclusions were reached because Ullrich didn’t evaluate the effects of hypoxia by itself and use different cell models and oxygen concentrations. Therefore, it still remains unknown about the role of hypoxia regulating the OTM process alone and synergetically because of lacking more evidences using standard experimental parameters. And the immune system may be an important mechanism involved in hypoxia-induced bone metabolism.

#### Autophagy

Autophagy is recently considered as a protective mechanism preventing excessive compression and hypoxia, and appears to participate in the degradation of OCs, OBs, and osteocytes. Autophagy is evolutionarily conserved to make sure the dysfunctional organelles are degraded in cells, stimulated by mechanical stimuli or local nutritional hunger. The degraded materials are encapsulated by the autophagosomes, which are double-membrane vesicles and coalesce with lysosomes to form autolysosomes. After the catabolism of autolysosomes, the degradation products are carried back to the cytoplasm for cyclic utilization. Autophagy normally occurs in cells at low levels, but during OTM, autophagy and relative proteins including Sequestosome1, Beclin-1, and microtubule-associated protein 2 light chain 3 were activated and increased in PDLCs under compressive force in vitro and in vivo^89,90^. It was reported that autophagy of PDLCs stimulated by compressive force negatively regulated osteoclastogenesis by inhibiting RANKL/OPG signaling in vitro^91^. The increase of autophagy in PDLCs can reduce the decline of bone density, inhibit the expression of inflammatory factors, and arrange the periodontal ligament during OTM in vivo^92^. However, another study highlighted the fact that osteocyte autophagy was activated under compressive force using the murine OTM model, and both in vitro mechanical compression and chemical autophagy agonist increased the secretion of RANKL in osteocytes by 3-fold and 4-fold respectively^93^. This phenomenon indicated that PDLCs and osteocytes may play different roles in tissue remodeling during OTM.

### Step 3: cell activation and differentiation

The cell strains on PDLCs and osteocytes directly induce the activation and differentiation of OCs and OBs, the direct regulator of bone formation and resorption. The knowledge of OB/OC differentiation mechanisms is the basis for understanding the whole regulatory mechanisms because once a force is applied, PDLCs, osteocytes, OBs, OCs, and other cellular components such as immune cells immediately constitute a huge and complex network for modulating the balances of osteogenesis and osteoclastogenesis. This network mainly consists of the PDLCs–osteocytes signaling, the PDLCs–OBs/OCs signaling, the osteocytes–OBs/OCs signaling, and the osteoimmunology system. The cell-to-cell communications of this network are mediated by the secreting proteins including various cytokines via autocrine or paracrine.

#### Differentiation and recruitment of osteoclast

Osteoclastogenesis is the OC differentiation process which is composed of four main phases: colony-forming unit-monocyte, monocyte, mono-nuclear OC, and multinuclear OC. Colony-forming unit-monocyte is a progenitor originating from the pluripotent hematopoietic stem cell. It resides in the bone marrow and could further transform into a monoblast, which in turn gives rise to the monocyte. Upon the release of monocytes into the bloodstream, they migrate to the bone tissue and differentiate into mononuclear OCs, which eventually fuse to form multinuclear OCs^94^. Osteoclastogenesis is predominantly regulated by two factors, M-CSF and RANKL-RANK-OPG system. Specifically, M-CSF, mainly detected in OBs and fibroblasts, triggers its receptor M-CSFR and subsequently induces the MAPK phosphorylation cascade^95^.

**Fig. 4:**
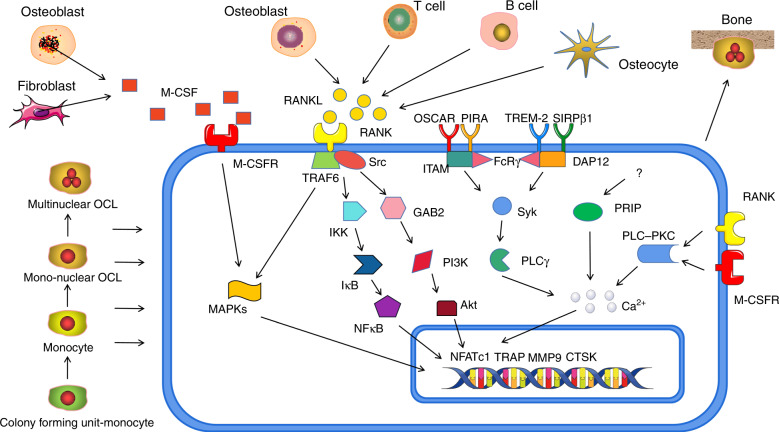
Differentiation and its mechanisms of osteoclast

RANKL, located on or cleaved from the cell membrane of OBs, osteocytes, T cells, and B cells, performs its effects mainly through the NFκB pathway^96^. It was reported that most of the OTM and formation of OCs were blocked in the RANKL-deletion mice, indicating the dominant role of RANKL produced by PDLCs and bone lining cells in osteoclastogenesis during OTM^97^. RANKL is believed to bind to both OPG, inhibiting its function, and to RANK, inducing osteoclastic differentiation. RANKL primarily induces its co-receptor RANK, initiating the intracellular recruitment of TNF receptor-associated factors (TRAF), among which TRAF6 plays the most critical role. TRAF6, in turn, acts through the inhibitor of NFκB (IκB) which generally sequesters and inhibits the NFκB transcription and the IкB kinase (IKK) which modulate phosphorylation of IκB^98^. OPG, another product of OB and B cell, is a member of the TNF receptor superfamily with 380-amino acid, acting as a decoy receptor for RANKL through its four cysteine-rich domains^99^. Upon activation, NFκB drives the expression of NFATc1, the major modulator of osteoclastogenesis, and consequently enhances transcription of OC differentiating markers including cathepsin K (CTSK), MMP9, and tartrated resistant acid phosphatase (TRAP)^100^. In addition, RANK signals to GRB2-associated binding protein 2 (GAB2) and Src family kinase, to activate PI3K/Akt signaling^101^. Notably, both M-CSFR and RANK could mediate NFATc1 via the activated PLC-PKC pathway and increased intracellular Ca^2+^ concentrations^102^. PLC-related, but catalytically inactive protein (PRIP) was previously identified as a novel inositol 1,4,5-trisphosphate-binding protein with a domain organization similar to that of PLCδ but lacking phospholipase activity. PRIP stimulates osteoclast differentiation through calcium-calcineurin-NFATc1 signaling via regulating intracellular Ca^2+ ^^103^. However, its upstream regulators were unclear. Additionally, some other co-stimulatory pathways regulating Ca^2+^ release were found. These pathways are mediated by the OB-associated immunoglobulin-like receptor (OSCAR), paired immunoglobulin-like receptor A (PIRA), triggering receptor expressed on myeloid cells 2 (TREM-2), and signal-regulatory protein β1 (SIRPβ1)^104^. OSCAR and PIRA bind to intracellular Fc receptor gamma chain (FcRγ), while TREM-2 and SIRPβ1 interact with DNAX-activating protein 12 (DAP12)^105^. FcRγ and DAP12 both contain immune receptor tyrosine-based motifs (ITAM), which are phosphorylated and activate spleen tyrosine kinase (Syk) and phospholipase Cγ (PLCγ) in turn, which induces the release of Ca^2+^^106^. The process discussed above is shown in Fig. 4.

#### Cytokines involved in osteoclastogenesis

Members of the IL family play major roles in the osteoclastogenesis regulations. IL-1, produced by macrophages in α and β forms, has been demonstrated as capable of stimulating the c-Fos and NFATc1 expression in OC through the MAPK/ERK signaling^107^. IL-1α was found to be one of the most abundant cytokines on the compressed side during the initial stages of OTM, while IL-1β expression significantly increased from the 7th day to the 14th day^108^. Compressive force and IL-1ß induced overexpression of COX-2 gene expression in hPDLCs in vitro^109^. Recently, exosomes from PDLCs stimulated with cyclic stretch suppressed IL-1β production by macrophages, indicating the important role of IL-1 in cell-to-cell communication in the PDL under mechanical loading. And IL-1 may be a great target for clinical intervention^110^. In-vitro and in-vivo studies have demonstrated that IL-6 could be produced by OBs and fibroblasts in periodontal tissues, inducing bone resorption alone and in concert with other bone-resorbing agents at the early phase of OTM within 24 h^111^. Glycoprotein 130 (gp130) is the central player of the receptor complex formed by IL-6–type cytokines during the activation of the IL-6 signaling pathway. It plays an important role in the formation of IL-6 binding sites by associating with the IL-6/IL-6R complex in the transduction of the IL-6 signal. Janus kinase (JAK) activation by gp130 results in activation of the signal transducers and activators of transcription STAT1 and STAT3 and the SHP2/Ras/MAPK signaling pathway. Liu et al observed enhanced expression of IL-6 and its key signaling factors gp130, STAT3, and SHP2 protein and mRNA at the tension and compression sides of the teeth in a mice OTM model, indicating the special role of IL-6 in the bone remodeling process^112^. IL-8 is secreted by monocytes and its expression induced by the initial orthodontic force on the first day significantly increased in the tension side, stimulating RANKL expression to regulate bone resorption^113^. The combination of mechanical vibration and compressive force upregulated RANKL/OPG, COX2/PGE2, IL-6, and IL-8 mRNA, and protein expression in isolated PDLCs, indicating the synergistic effect of several inflammatory factors during OTM^114^. Orthodontic forces also resulted in increased levels of IL-17 and IL-23 in the gingiva crevicular fluid (GCF), which were statistically significant at 7 days of force application at compression sites in orthodontic patients^115^. However, we do not know the exact effect on them.

Tumor necrosis factor (TNF), primarily produced in α and β forms by monocytes, macrophages, and OBs, can directly bind with the TNF receptor-1to induce RANK expression of OC precursors, and induce RANKL expression of osteocytes by increased sclerostin expression^116,117^. Several cytokines have interaction effects with TNF and collectively constitute a cytokine regulatory network of TNF-induced osteoclastogenesis. For example, TNF-α enhanced IL-6 and IL-1 expression in hPDLCs which in return enhanced the activities of TNF-α, forming a positive feedback loop^118^.

Chemokines, classified into four subfamilies depending on whether the first two cysteines near the N-terminal are separated (CXC, CX3C) or not (CC, C), are essential signals for the chemotaxis and localization of circulating hematopoietic cells into tissues. They are synthesized by many cell types including fibroblasts, stromal cells, endothelial cells, bone cells, mast cells, and leukocytes. Chemokines interact with their receptors to form a complex network relationship, that is, a chemokine can bind to multiple receptors (CCR), and a receptor can also have multiple chemokine ligands (CCL). CCR1 is expressed in marrow cells and binds to CCL3, CCL5, CCL-7, and CCL9, which are produced by OCs and OBs, and markedly increased by IL-1α and TNF-α in OBs. All the chemokines directly stimulated the chemotactic recruitment OC formation in marrow cultures through a pathway dependent on the presence of RANKL and were diminished in the CCL3^−/−^ mice during OTM^119^. In addition, the CCR2-CCL2 axis is positively associated with the recruitment and formation of OCs during OTM, but the mechanism is still unclear^120^. On the contrary, CCR5 seems to inhibit OC formation in OTM because CCR5-deficient mice have a much higher rate of OTM and increased numbers of OCs. At the molecular level, in CCR5^−/−^ mice, OB differentiation markers (Runx2 and OC) and negative OC differentiation regulators (IL-10 and OPG) were significantly decreased compared to wild type, while cathepsin K, RANKL, and MMP13 were significantly higher^121,122^. Recently, therapeutic strategies based on the increase of Atypical chemokine receptor 2 (ACR2), a decoy receptor for CC chemokines expressed in OCs and OBs, might be useful to inhibit bone resorption^123^. Therefore, chemokines and their receptors are potential therapeutic targets in the future.

#### Activation and differentiation of osteoblast

**Fig. 5:**
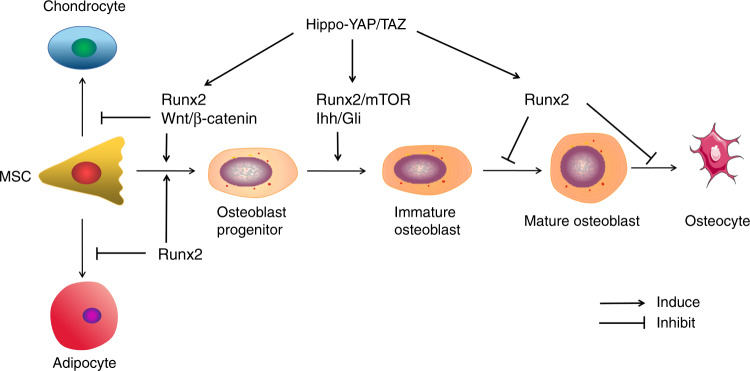
Regulation of osteoblast differentiation

After that PDLCs sense the mechanical stimuli, MSCs are activated to differentiate into OBs, which specifically express osteocalcin and Runx2. MSCs firstly differentiate into OB progenitors and immature OBs. The immature OBs, which express bone matrix protein (BMP) genes and high levels of OPN, differentiate into mature OBs, which express high levels of osteocalcin. Finally, the mature OBs transform into osteocytes after being embedded in the bone matrix. Runx2/mammalian target of rapamycin (mTOR), Indian Hedgehog (Ihh)/Gli, Wnt/β-catenin, and Hippo/Yap pathways are the most essential pathways for OB differentiation (Fig. 5).

Runx2 is classified by different N-termini into two isoforms: the type I Runx2 transcribed from the proximal promoter and type II Runx2 transcribed from the distal promoter. Both Runx2 types have similar functions in chondrocytes and OBs. Runx2 inhibits MSCs’ differentiation into chondrocytes and adipocytes and directs them to preosteoblasts^124^, which then activates the gene expression of major ECM protein including the alkaline phosphate (ALP), Col1a1, OPN, bone sialoprotein, and osteocalcin^125^. However, overexpression of Runx2 severely inhibits OB maturation and the differentiation into osteocytes, maintaining a supply of immature OBs^126^. Many molecules interact with Runx2 and regulate its functions. For example, mTOR is regulated by Runx2 to phosphorylate Akt for modulating cell proliferation and differentiation in OBs and BMSCs on the tension side during OTM in vivo and in vitro. Aonuma reported that Runx2(+/-) mice exhibited suppressed mTORC2/Akt activity^127^. P70S6 K as a downstream molecule of mTOR is activated by phosphorylation and subsequently promotes the synthesis of ribosomal and translational proteins. The expression of PI3K, Akt, and P70S6K in human periodontal tissues during OTM began to increase at 3 days, indicating the PI3K/Akt/mTOR/P70S6K signal pathway was involved in OTM^101^. The role of mTOR pathways participating in the regulation of osteogenesis worth further study and it may be an important target for pharmacological intervention.

Ihh binds to its membrane receptor Patched and relieves the repression of another receptor Smoothened, ultimately regulating the transcription factor Gli. Ihh conditional knockout mice using Prrx1 promoter Cre transgenic mice, in which Cre is expressed in mesenchymal cells in the limbs and calvaria, obtained impaired bone formation and suppressed Runx2 expression phenotype in the limbs. Therefore, Hh signaling is required for OB development. A study firstly found Gli1+ cells expressed in PDL which were proliferated and differentiated into osteoblastic cells under tensile force and both pharmacological and genetic Gli1 inhibition led to arrest of bone remodeling. Furthermore, Yap expressed in Gli1+ cells and decreased after the suppression of Gli1+ cells. Conditional ablation of the Yap gene in Gli1+ cells inhibited the bone remodeling as well, suggesting Gli1+ cells are force-responsive cells^128^. Whether the Ihh/Gli pathway takes part in the OB differentiation and bone remodeling during OTM remains investigated.

The Wnt/β-catenin signaling pathway also plays a vital role. Wnt3a, Wnt10b, and Wnt5a were found to be involved in the pathway during OTM^129,130^. The Wnt receptor Frizzled and Wnt coreceptors lipoprotein receptor-related protein 5 (LRP5) and LRP6 collectively form a ternary receptor unit at the cell membrane. Stimulating signals activate the T-cell factor and transcription factors lymphoid enhancer factor via the unit, upregulating osteogenic genes. The β-catenin completely eliminates the potential of OB progenitors for differentiating into chondrocytes, further direct the OB progenitors into immature OBs^131^.

A recent high-throughput sequencing analysis study suggested that the Hippo signaling pathway also plays a vital role in the mechanical signal transduction because several important components of the Hippo signaling pathway, including YAP1, WWTR1, TEAD2, CTGF, DVL2, GDF5, GLI2, LIMD1, WTIP, LATS1, and TEAD1, were significantly upregulated in stretched PDLCs^74^. YAP and TAZ are transcriptional coactivators activated by the translation of physical cues into biochemical reactants through Hippo signaling. After they are shuttled into the nucleus, they could interact with the TEA domain (TEAD) family transcription factors to regulate a broad spectrum of downstream genes. YAP, TAZ, and RUNX2 expression started increasing at 2d in the PDLCs in the rat OTM model and TAZ expression was associated with RUNX2 expression^132^. Cyclic stress could significantly increase the expression of YAP target genes such as CTGF and CYR61, and the nuclear translocation of YAP in the PDLCs. Furthermore, knockdown of YAP suppressed the cyclic stretch-induced osteogenesis in hPDLCs, while overexpression of YAP enhanced osteogenesis^133^. Expression of TEAD1 also highly correlated with that of OPG and decreases in response to mechanical force in human PDLCs. Knockdown of TEAD1 downregulated expression of OPG and promote osteoclast differentiation. Mechanical force-induced decreased binding of TEAD1 on OPG promoter. OPG was also affected by pharmaceutical disruption of the Hippo signaling pathway^134^.

In a word, the role of the Hippo pathway in regulating OB and OC differentiation needs research in detail.

#### Cytokines involved in osteogenesis

Growth factors are biologically active polypeptide hormones affecting the immune function as well as the development of PDL or bone cells by binding to specific cell surface tyrosine kinases receptors on OBs and fibroblasts. For instance, PDGF was found to stimulate collagen synthesis of PDLCs and enhance the OB proliferation in vitro without effect on the differentiation^135^. TGF-β1 combined with PDGF-BB enhance the expression of integrin β3 in the periodontal membrane and accelerates periodontal remodeling^136^. Insulin-like growth factor (IGF)-1 exerts mitogenic effects on fibroblasts in a dose- and time-dependent manner without affecting cell adhesion, migration, and expression of type I collagen^137^. Fibroblast growth factors and epidermal growth factor also promote the proliferation of PDLCs and OBs in a dose-dependent manner but have no effect on cell differentiation^138^. In conclusion, growth factors mainly operate the proliferation promotion effects rather than the differentiation promotion effects and have the potential for clinical application in the acceleration of OTM and enhancement of retention.

It is worth noting that parathyroid hormone (PTH) and parathyroid hormone-related protein (PTHrP), sharing the common receptor PTHR1, can regulate bone remodeling both by osteogenesis or osteoclastogenesis. It is well-known that intermittent use of PTH/PTHrP shows predominant anabolic effects on bone tissue, while continuous use of PTH/PTHrP leads to catabolic effects^139^. The mechanism is that transient combination between PTH/PTHrP and PTHR1 activates several pathways including PKA, PKC, and others that control osteoblastogenesis, while prolonged combination activates prolonged cAMP production that is thought to cause increased bone resorption^140^. Therefore, PTH/PTHrP were used for accelerating bone remodeling rate during OTM and the newest study proved that local injection carried by PTH/PTHrP controlled release hydrogen both enhanced OTM rate and reduced the relapse rate^141^.

#### PDLCs–OBs/OCs signaling

Subjected to orthodontic forces, PDLCs are activated and regulate OB and OC via the expression of some regulators, which means the existence of PDLCs–OB/OC signaling. For example, a study recently found that PDLCs under static compressive force in vitro for up to 6 days showed significant upregulation of cFOS and down-regulation of RUNX2, for inducing bone-resorption activities^142^. The transcription factor c-Fos controls the differentiation of osteoclasts and is expressed in PDLCs after mechanical stimulation in vitro. Overexpression of c-Fos by employing c-Fos transgenic mice accelerated tooth movement without causing more root resorption^143^. Therefore, these key regulators mediating cell-to-cell communication are great targets for the intervention of OTM.

The PDLCs–OB signaling often includes the expression of bone-formation stimulative or inhibitory factors such as wnts, GDF15, ephrin, asporin, and periostin. OTM was found to significantly stimulate the Wnt1 expression on the tension side on day 5, whereas the expression on the compression side did not change. This increase in the Wnt1 expression, shown in vivo, was also noted after the application of static tensile force in vitro both in PDLCs and long bone osteocytes. In contrast, a 2.4 g/cm^2^ compressive force led to the attenuation of the Wnt1 gene expression in a force-dependent manner, although it was much lower than a normal orthodontic force^144^. The TGF-β/BMP-family member GDF15 is well-known for its important functions in the regulation of cell metabolism in response to cellular stress. Symmank et al detected enhanced expression levels of GDF15 in the rat OTM model as well as in mechanically stressed hPDLCs. Moreover, the stimulation of human primary osteoblast with GDF15 in vitro resulted in increased transcription of osteogenic marker genes like RUNX2, OCN, and ALP^145^. Recently, Ephrin ligands and Eph receptors, which are tightly connected with alterations of the cytoskeleton, have recently been shown to be involved in the regulation of bone homeostasis. It was reported that activation of the ephrinB2/ EphB4 signaling on OBs led to stimulation of bone formation while the activation of ephrin-A2/EphA2 signaling on OBs inhibited the activation of osteoblast-specific gene expression, leading to bone resorption. Diercke et al. stated that static compressive forces significantly induced the expression of ephrin-A2 on PDLCs, while the expression of ephrin-B2 was significantly down-regulated, establishing a role for this ligand/receptor system linking mechanical forces and cellular reactions^146^. Asporin, which is also called periodontal ligament-associated protein 1, was found to increase in PDLCs on the compression side in vivo and under compressive force in vitro. Asporin directly binds to BMP-2 and leads to the bone formation inhibition via suppressing TGF-β/Smad signaling^147^. How the mechanical force regulates the expression of aspirin in PDLCs has not been understood yet. Periostin is a matricellular protein which is a critical role in resisting occlusal loading and maintaining PDL integrity. Tensile stress upregulated periostin expression in mouse PDL and hPDLCs during OTM, and periostin promoted type I collagen and α-SMA expression levels in hPDLCs^148^. Deletion of the periostin caused the decrease of TRAP-positive cells and impaired integrity of collagen fibrils^149^. But it remains unclear that whether this mechanism is through a direct or an indirect way during OTM.

On the other hand, PDLCs are facilitators of OC formation and recruitment through the cell-cell-mediated pathway. First, fibroblasts attract OC precursors and bind them with intercellular adhesion molecule-1 (ICAM-1) to leukocyte function-associated antigen-1 (LFA-1) on the OCs^150^. Second, osteoclastogenesis-stimulatory molecules such as M-CSF, TNF-α, and RANKL are upregulated in fibroblasts. Third, fibroblasts retract and TRAP-positive OC precursors migrate on the bone surface, forming multinucleated OCs^151^. However, the expression of OPG is found up to thousands-fold higher than RANKL. This phenomenon can be explained that the tight cell–cell contact between fibroblasts and OCs could create a favorable micro-environment for RANKL–RANK binding, preventing the function of OPG and stimulating explosive osteoclastogenesis effects. Therefore, the overexpression of OPG is needed and prevents OC formation at sites where this is not wanted^150^.

#### Osteocytes–OBs/OCs signaling

Under tensional loading, osteocytes produce anabolic molecules including wnts and NO^144^. NO is produced by inducible nitric oxide synthase (iNOS) or endothelial nitric oxide synthase (eNOS) in osteocytes and OBs, demonstrated as an early mediator of bone remodeling. The eNOS are characterized by low-output NO production to maintain hysiological function, while iNOS is upregulated during inflammatory processes and is responsible for producing high amounts of NO^152^. It was found that eNOS-positive osteocytes increased in the tension area while iNOS-positive has no change after force application, and in the compression area, both eNOS-positive and iNOS-positive osteocytes increased, suggesting that eNOS enhances bone formation in the tension area, while iNOS enhances inflammation-induced bone resorption in the compression area^153^.

Osteocytes generally regulate OC formation and activation by secreting osteoclastic molecules mainly including RANKL and IL-6. Osteocytes were regarded as the critical source of RANKL in alveolar bone remodeling during OTM because osteocytes expressed a much higher amount of RANKL than other cells did in periodontal tissue. The critical role of osteocyte-derived RANKL was confirmed by the reduction of OTM in mice specifically lacking RANKL in osteocytes^154^. It was reported that recombinant IL-6 and IL-6 receptors enhanced the expression of RANKL via JAK2 and STAT3 in osteocytes and osteocytes significantly stimulated osteoclastogenesis when co-cultured with osteoclast precursors^155^. However, whether the in vivo source of IL-6 is PDLCs-dependent remains unknown.

In conclusion, there are not enough studies regarding to the osteocytic functions during OTM, especially the 3D osteocyte model which can mimic the alveolar bone and the co-culture model of osteocytes and PDLCs, under an adequate force with a magnitude closing to orthodontic force.

#### PDLCs–osteocytes signaling

As the mechanical sensor cells, PDLCs and osteocytes do not regulate OC/OB differentiation independently, but synergistically through a communication network mediated partly by sclerostin. The sclerostin (encoded by SOST) is a Wnt antagonist that exhibits increased expression on the compressed side during OTM and interacts with LRP5/6 to suppress the binding of the Wnt receptor and inhibit bone formation^156^. In an osteocyte–PDLCs coculture system designed to mimic OTM, compressive force modulated the SOST expression in the isolated human PDL and thereby upregulating osteocytic SOST via paracrine activation. This system did not affect the RANKL or OPG expression in osteocytes, suggesting that the bone resorption pathways are acted upon in a PDL-dependent and osteocyte-independent manner through RANKL/OPG signaling. Moreover, sclerostin neutralizing antibody significantly attenuated the upregulation of SOST that was induced by compressive force^157^. In another osteocyte–PDLC coculture system, recombinant sclerostin attenuated Wnt1 in PDLC, whereas the antisclerostin antibody upregulated its gene expression, indicating that mechanically driven Wnt1 signaling in PDLC might be regulated by osteocytic sclerostin^144^. These results collectively indicated the existence of PDLCs–osteocytes–OBs crosstalk, which is an important cellular signal network response to orthodontic forces and is mediated by the sclerostin and wnt proteins.

#### Immune cells

As we discussed above, cell strains induced by orthodontic force, along with hypoxia and cell autophagy, may synergistically regulate PDL and bone remodeling by inducing an aseptic inflammatory response. It is now evident that the immune systems and skeletal are functionally linked and share common cells and signaling molecules such as cytokines, which is called osteoimmunology firstly by Arron and Choi in 2000^158^. While it is known that PDLCs and osteocytes are critically involved in the biological regulation of OTM by a mechanically triggered release of cytokines, the role of immune cells secreting inflammatory factors is gaining increasing attention in recent years, including macrophages, T cells, and B cells. Macrophages are mainly classified into M1 type and M2 type. M1-type macrophages can release various inflammatory factors such as IL-1, IL-6, and TNF-α, and initiate osteoclastogenesis. M2-type macrophages mainly secret TGF-β and IL-10, and inhibit the formation of OCs and support bone deposition^159^. In the early stage of OTM, macrophages are recruited to the inflammatory site, and various inflammatory factors promote their polarization toward the M1 type on the compressed side. At the same time, M1-type macrophages promoted the expression of TNF-α to accelerate OTM^160^. The M2-type macrophages are not significantly recruited until the late stage of OTM, which is crucial for the cessation of bone resorption and the beginning of tissue repair^161^. There are few studies on the specific signaling pathways that affect macrophage polarization, which need to be solved. Activated T cells are suggested to induce OCs differentiation by expressing RANKL. However, two kinds of T-helper cells: Th1 cells that secrete IFN-γand Th2 cells that secrete IL-4, are found to inhibit OC activities^162^. This discrepancy can be explained by the new T-helper cell subtype, Th17 cells that produce IL-17 and promote osteoclastogenesis, whose number was positively correlated with RANK expression and numbers of osteoclasts in rat OTM model^163^. PDLCs did not express IL-17 under compression, indicating that the Th17 cells are needed to be recruited to the bone resorption site^164^. B cells also play important roles in osteoclastogenesis by expressing RANKL, IL-6 and TNF^165^. But we don’t know how these immune cells sense the external mechanical signals and response. Ogawa et al. determined the individual contribution of each cell type to osteoclastogenesis during OTM as a result of being targeted by TNF-α and results suggested that T cells had no contribution while stromal cells and osteocytes contributed more than bone marrow macrophage in osteoclast formation during OTM^166^. So the immune cells may mainly play auxiliary roles in inflammation trigger and the mechanical signal sensors PDLCs and osteocytes are the most important cells inducing the aseptic inflammatory response, secreting inflammatory factors such as cytokines and thus regulating osteogenesis and osteoclastogenesis.

### Step 4: tissue remodeling

#### Tissue remodeling of PDL

In response to different loadings, followed by different mechanosensory approaches, intracellular signals and gene expressions, PDLCs, OBs and OCs are believed to secrete catabolic and anabolic bioactive molecules for both tissue resorption and tissue formation, finally accomplishing tissue remodeling. In the compression side, PDL is degraded to create tooth movement space while new PDL tissue is simultaneously formed to maintain the attachment. A compressive strain was found to decrease collagen 1 mRNA, type I collagen, and increase MMP2 mRNA levels in PDLCs^167^. After the attachment of the PDL fibers to the bone is lost, the non-functional type I collagen fibers are degraded and replaced by type III collagen to contain a loose connection. The number and activity of OCs are improved via OPG/RANKL ratio modification in this process, along with the increased activity of PDLCs. In the tension side, PDL remodeling takes place after the fibers are stretched. New PDL matrix contains type I collagen fibers is formed to maintain the PDL width and the attachment of the alveolar bone to the tooth^167^.

#### Tissue remodeling of bone

In the compression side, before the actual bone resorption can occur, OBs have to degrade the osteoid through MMP activity, aiming to make the differentiated OCs attach to the bone surface. The attached OCs to the bone surface undergo morphological changes and then actual resorption takes place at the ruffled border, where OCs release hydrogen ions to dissolve the anorganic matrix and enzymes such as MMPs and cathepsins to resorb organic matrix in bone. Cathepsins and MMPs were found to increase at the compression site in 3 h to 1 week of OTM and decrease towards the second week in vivo^168^. Bone formation tends to be slower than bone resorption, which possibly explains the radiographic widening in PDL observed during OTM. In the tension side, OBs are responsible for the formation of new bone by firstly producing new ECM and then mineralizing them, while some OBs will be entrapped in the bone and turn into osteocytes. In an in vitro study, human PDLCs in response to tensile strains increasingly expressed osterix, bone sialoprotein, OC, ALP, type I collagen, and BMPs, which benefit to bone synthesis and tissue remodeling^169^.

#### Tissue remodeling of tooth

When heavy orthodontic forces are applied over a sustained period of time, hyalinization of the compressed PDL may rapidly develop. External apical root resorption is initiated when the protective layer of cementoblasts undergoes apoptosis and enables odontoclasts to resorb cementum and dentin^170^. The differentiating, morphologic, and functional characteristics of odontoclasts are extremely similar to those of osteoclast^171^. Odontoclasts can be activated by RANKL, MCSF, IL-1, IL-6, and TNF-α, and inhibited by OPG^172^. Proper adhesion of the odontoclasts to the mineral substrate enables activation of all the intracellular machinery necessary to degrade the mineral component. Odontoclasts attachment to the mineral surface is mediated through integrins and ECM proteins^172^. Resorption also follows a similar process in bone and dentin. Gene microarray analysis found greater overexpression of genes associated with cell fusion (CD9), cytoskeleton (β-actin, actinin, filamin, and tubulins) and catabolic genes (RANK and TRAP) in clastic cells cultured on dentin substrate as compared with bone^173^. It is worth noting that cementum is commonly regarded as an antiresorptive barrier because it lacks a mineral-remodeling process. At the transcriptomic level, cementocytes have shown an in vivo expression profile similar to that of the osteocytes, being able to express dentin matrix protein 1, sclerostin, OPG, and RANKL^174^. Moreover, cementocytes express significantly higher levels of OPG and lower levels of RANKL mRNA than osteocytes from the alveolar and long bones, indicating a protective effect that inhibits cementum resorption or remodeling^175^. To prevent orthodontic root resorption, approaches mainly via inhibiting odontoclast activities or enhancing cementocyte activities may be useful.

### Clinical implications

#### OOF

The term “OOF” has been elucidated as the lightest force that produces the most rapid tooth movement with the least tissue damage and the most patient comfort^176^. The magnitude of orthodontic force is associated with adverse effects including uncontrolled tipping, increased hyalinization, root resorption, and even tooth exfoliation. Commonly, the low-magnitude forces are preferable because the heavy force increased the risks for root resorption and hyalinization by inducing sharp up-and-downs of cytokine levels and apoptosis of cementoblasts^177^. However, the appearance of hyalinization is an important component affecting tooth movement rate, but a clear relationship between force-related variable and extent of hyalinization has not been found yet. Hyalinization occurred even with a force as low as 5 cN. Compared with heavy continuous forces provided by fixed appliances, interrupted light forces derived from Invisalign caused fewer apical root resorption in orthodontic patients^178^. The heavy tensile force also inhibits bone formation via decreasing expression of Runx2 in osteoblast-like cells isolated from fetal rat calvariae^179^. According to Alikhani et al, a saturation of the biological response was observed in a rat OTM model upon a certain magnitude of force, and higher forces introduced no effects on the tooth movement^180^. Therefore, in a biological opinion, the OOF may be the lightest force that can initiate OCs activation and further tissue resorption. However, evidences have not reached a consensus about the exact magnitude of OOF.

Evidently, the exact range of optimal orthodontic forces has not come to a conclusion because multiple factors of previous in-vivo or in-vitro studies could contribute to the inconsistency. Firstly, at the cellular level, it is known that the stiffness of ECM plays a direct role in regulating multiple cellular functions and intracellular signaling. The differences of matrix stiffness between in-vitro models and real PDL lead to the inaccurate results of some studies, regardless of the 2D or 3D models. As we described above, the elastic modulus of porous PLGA is about 4 MPa, much higher than that of collagen gel and very close to that of human PDL. It was indicated that the RANKL mRNA expression of PDLCs was significantly up-regulated by compressive force ≥25 g/cm^2^ in the 3D model using PLGA. Force about of 25 g/cm^2^ equals blood pressure in PDL terminal capillaries and should be optimal for OTM^10^. However, in clinical application, we do not know the forces applied on cells because of the loss in the process of force transfer during OTM. All components of the orthodontic fixed appliance, including wire, bracket, and adhesive, seem to influence, to some extent, the biomechanics of OTM^181^. Secondly, at the histological level, during different types of tooth movement, the area of PDL participating in a direct load of mechanical force is different. For example, Wu et al used a finite element model and pointed out that optimal tipping forces ranged 28–32 g for labial-direction and 40–44 g for distal-direction in a maxillary canine, while the optimal forces for translational motion were 110–124 g for labial-direction and 130–137 g for distal-direction^182^. Thirdly, at the individual level, optimal orthodontic forces varied in individuals since genetic differences, variation in patient periodontal condition, degree of aging and alteration of hormonal readiness could all lead to diversity and thereby required personalized treatment. The rate of OTM still varied among and within individuals even with standardized, constant, and equal forces^183^. A personalized OOF may be used for different groups of patients, and will be based on the precise measurement of PDL area and matrix stiffness in single teeth.

Recent studies also indicated the existence of a circadian rhythm of the osteogenic factors within periodontium during OTM. It was suggested that cultured hPDLCs express circadian clock genes and genes associated with bone and periodontal remodeling are influenced by the circadian rhythm^184^. The osteogenic genes’ expressions as well as the protein releases also sustained a circadian oscillation trend in vivo^184^. Intracellular Ca^2+^-regulated biorhythm might regulate the response of osteocytes to mechanical stimuli by controlling the spatiotemporal pattern of sclerostin expression via the osteocytic network^185^. It highlights the importance of the precise timing of force loading and a periodicity pattern of orthodontic traction at night in further orthodontic treatment. However, there are only limited evidences and whether the circadian rhythm really exists during OTM is still a question.

### Acceleration of OTM

Osteoclast activity and bone resorption activity on the compression side are considered to be the rate-limiting step that determines the speed of OTM. Attempts to accelerate OTM should be focused on activity of osteoclast and bone resorption. Therefore, based on the four steps of OTM, potential targets involved in OC regulation may be useful for the objective of controlling OTM rate. For example, the sympathetic nervous system regulates bone resorption through Adrb2 and the injection of nonselective Adrb2 agonist accelerated OTM^27^. Local injection of sclerostin protein in the alveolar bone at the compression side also accelerated OTM in rats by promoting osteoclastogenesis^5^. Other key regulators mediating this network are waiting to be explored and their application may benefit the clinics. Among the vast targets we included in the review, RANKL and OPG are most important regulators for OC differentiation. However, the use of exogenous RANKL to accelerate OTM has not been attempted to date, because large quantities of RANKL for systemic therapy may subsequently cause serious systemic loss of skeletal bone, while local therapy may only affect several tooth. The controlled and sustained local release of RANKL from a NF-hydrogel carrier matrix was introduced and maximized its therapeutic benefit whilst minimizing systemic side effects, which maybe benefits further studies^186^. The effectiveness of recombinant OPG fusion protein was also assessed and it effectively inhibited osteoclastogenesis resulting in improved bone quantity and orthodontic anchorage^187^. The combination of bone homeostasis regulators and new drug delivery systems or methods is a promising direction for clinical application.

Inflammatory markers also play an important role in OC recruitment and differentiation, such as cytokines. Thus, any attempt to increase inflammatory markers may increase the speed of OTM. The level of cytokines can be increased by locally injecting cytokines at the OTM site. Many researchers have conducted studies on the effectiveness of clinical injections since inflammatory markers have a very short half-life. Local injection of the platelet-rich plasma (PRP), which is defined as an autologous concentration of platelets contains growth factors cytokines, proteases and leukocytes, successfully accelerated OTM^188^. However, a study found that local injection of PRP did not have long-term acceleration effects, although it increased the rate of canine retraction initially^189^. The various molecules we discussed above provided a great number of targets for controlling OTM speed. Except for selecting an optimal target with maximal effects and minimal side effects, an adequate drug delivery approach with a location-specific characteristic and a long-time effect is also important.

It can also increase local inflammatory markers by causing microtrauma such as corticotomy, piezocision, and micro-osteo perforations (MOP), etc. The corticotomy-assisted orthodontic treatments disconnected the bone cortex without damaging the cancellous bone, blood vessels, nerves orperiosteum. It routinely consisted of buccal and lingual full-thickness flaps, subsequent partial decortication of the cortical plates, bone augmentation, and closure of primary flaps. Corticotomy accelerate OTM through inducting local osteoclastogenesis and stimulating macrophage infiltration^161^. Controversially, some recent studies have found that corticotomy has no effect on acceleration of OTM^190–192^. These inconsistent results and their sources should be clarified by systematically comparison of the different trail designs in different studies. Besides, MOP is a minimally invasive procedure without the need of reflecting a full-thickness flap and used as an alternative method to conventional corticotomy. According to research, MOPs significantly accelerated tooth movement by increasing the expression of TNF-α and promoting the proliferation and apoptosis of PDLCs^193,194^. Similarly, Alkebsi et al. had conducted randomized controlled trials, which indicated that MOPs were not able to accelerate the rate of OTM^195,196^. Piezocision is an optimized procedure using piezoelectric devices instead of scalpel and mallet. And piezocision-assisted flapless corticotomy was supposed to be effective in accelerating OTM^197^. According to available information, some systematic reviews indicated that piezocision results in acceleration of OTM, but high-quality evidence was required^198^. In a word, the effects of surgery-assisted orthodontic treatments tend to be uncertain and unstable, with unknown mechanisms.

There are also non-invasive methods to accelerate OTM such as low level laser therapy (LLLT) and vibration^199^. PDLCs response differently to these physical stimuli compared to general orthodontic forces. LLLT increased the levels of IL-1β and MMP-9 in GCF, decreased the levels of MMP-8, which may induce the proliferation of osteoblast and osteoclast and leads to remodeling processes in the alveolar bone^200^. Vibration promotes proliferation of osteoclast via NF-κB activation^201^ and upregulates the PGE2 and RANKL in the PDLCs^202^. But vibration is also a controversial method because some studies indicated that vibration had no effect on acceleration of OTM^203,204^. There are weak evidence suggests that stimulus of vibration is effective, which signifies that high-quality clinical trials are also needed^205^.

In summary, the acceleration effects of nearly all the existing methods are uncertain and controversial, due to some unknown factors. Illuminating the underlying mechanisms of each method may improve this condition.

### Prevention of root resorption

Although it seems that root resorption is ineluctable during OTM, there are varied methods to reduce root resorption, including pharmacological agents, microtrauma and noninvasive methods. Makrygiannakis et al. summarized existing research and the systematic review suggested that a comparative decrease of root resorption was noted after the administration of the alendronate, ibuprofen, growth hormone, low doses of meloxicam, simvastatin, lithium chloride and strontium ranelate, while no difference was noted for acetaminophen, aspirin, fluoxetine, atorvastatin, misoprostol, zoledronic acid and zinc. The quality of the available evidence was considered at best as low so that high-quality clinical trials are needed^206^. Surgery is used to accelerate OTM, but their impact on root resorption is inconclusive. Corticotomy and corticision have effect on reduction of root resorption during the early stages of OTM, but root resorption was increased with heavy force^207^. Moreover, most surgical methods including alveolar decortication, piezocision, and MOP increase the root resorption, and the degree of root resorption is positively correlated with the degree of surgical injury^208–210^, which indicates that the application of these surgical methods should be thought synthetically and prudently. It is also reported that the treatment with high-frequency mechanical vibration reduced root resorption effectively^211^ and LLLT contributed to the reduction of root resorption but was not statistically significant^212^. There is a process of root repair when orthodontic force is stopped. Thus, it is advisable to discontinue the orthodontic forces when the root resorption is obvious. Mehta et al. reported the rest period of 6 weeks showing good healing after active intrusion of orthodontic forces^213^. Compared with continuous force, intermittent force significantly reduced the amount of root resorption and avoid unwanted rotational movement, although it extends the time of treatment^214^. The problem how to specifically control the odontoclasts and cementocyte is waiting to be solved.

## Conclusion

In this review, we summarized the current knowledge and explained the hypothetic theory of the mechanisms underlying OTM mainly through classifying the whole biomechanical events into four steps and introduced the developments of current study models and clinical applications. We reached some important conclusions including the urgent need for standardized 3D-PDLCs and 3D-osteocytes in-vitro models, the possible study direction about the dependent and independent functions of PDLCs and osteocytes under OTM context and long bone context, the possible OOF both in researches and in clinics, the potential pathways for accelerating OTM and preventing root resorption. Therefore, more future studies are required and we hope that this review may provide assistance.

## Methods

### Protocol

The literature screening of this review followed the Preferred Reporting Items for Systematic Reviews and Meta-Analyses statement guideline^215^.

### Eligibility criteria

The inclusion criteria used in this review were as follows: articles, reviews, editorials, research letters, and systematic reviews that related to all the items we listed in the review including research models, biomechanical, and biological events during OTM, OOF, acceleration of OTM and root resorption. The exclusion criteria were as follows: case reports, abstracts; studies that not involving OTM mechanism.

### Information sources

A systematic search to identify all the relevant studies was conducted in the following four databases: MEDLINE (via PubMed), Embase, the Cochrane Library, and Web of Science. A supplemental manual search was also conducted by reviewing the reference lists of the related papers. The gray literature was searched on Clinicaltrial.gov, Open Grey, and the World Health Organization’s International Clinical Trial Registry Platform. All searches were conducted in December 2020, and the year of publication was restricted as from 2010 to 2020.

### Search

The search strategy included two keywords: “orthodontic” and “tooth movement”. The keyword “orthodontic” was expanded to “orthodontics” and “orthodontically”, and the abbreviation “OTM” were also concerned. The search strategy was as follows: (OTM[Title/Abstract]) OR ((tooth movement[Title/Abstract]) AND (((orthodontic[Title/Abstract]) OR (orthodontics[Title/Abstract])) OR (orthodontically[Title/Abstract]))), which was developed for MEDLINE and adapted for the other databases.

### Study selection

Two reviewers (Y.L. and Q.Z.) screened the titles and abstracts of the identified studies independently and in duplicate. Consensus was obtained by discussion and consultation with a third reviewer (M.B.) to resolve any disagreements during study selection and data extraction.

### Risk of bias in individual studies

The risk of bias of entitled studies was assessed by two independent dental investigators (Y.L. and Q.Z.). Arising disputes were discussed with M.B. The SYRCLE’s risk of bias tool was used for animal studies’ quality assessment^216^. The ROBIS tool was used for systematic reviews^217^ and the Cochrane ROB tool was used for randomized controlled trials^218^.

## Supplementary information

SI-Figure 1
