# Supplementary figures and images for: Biomechanical and biological responses of periodontium in orthodontic tooth movement: up-date in a new decade
Source: Int J Oral Sci. 2021 Jun 28;13:20. doi: 10.1038/s41368-021-00125-5 (PMC8239047; doi:10.1038/s41368-021-00125-5)

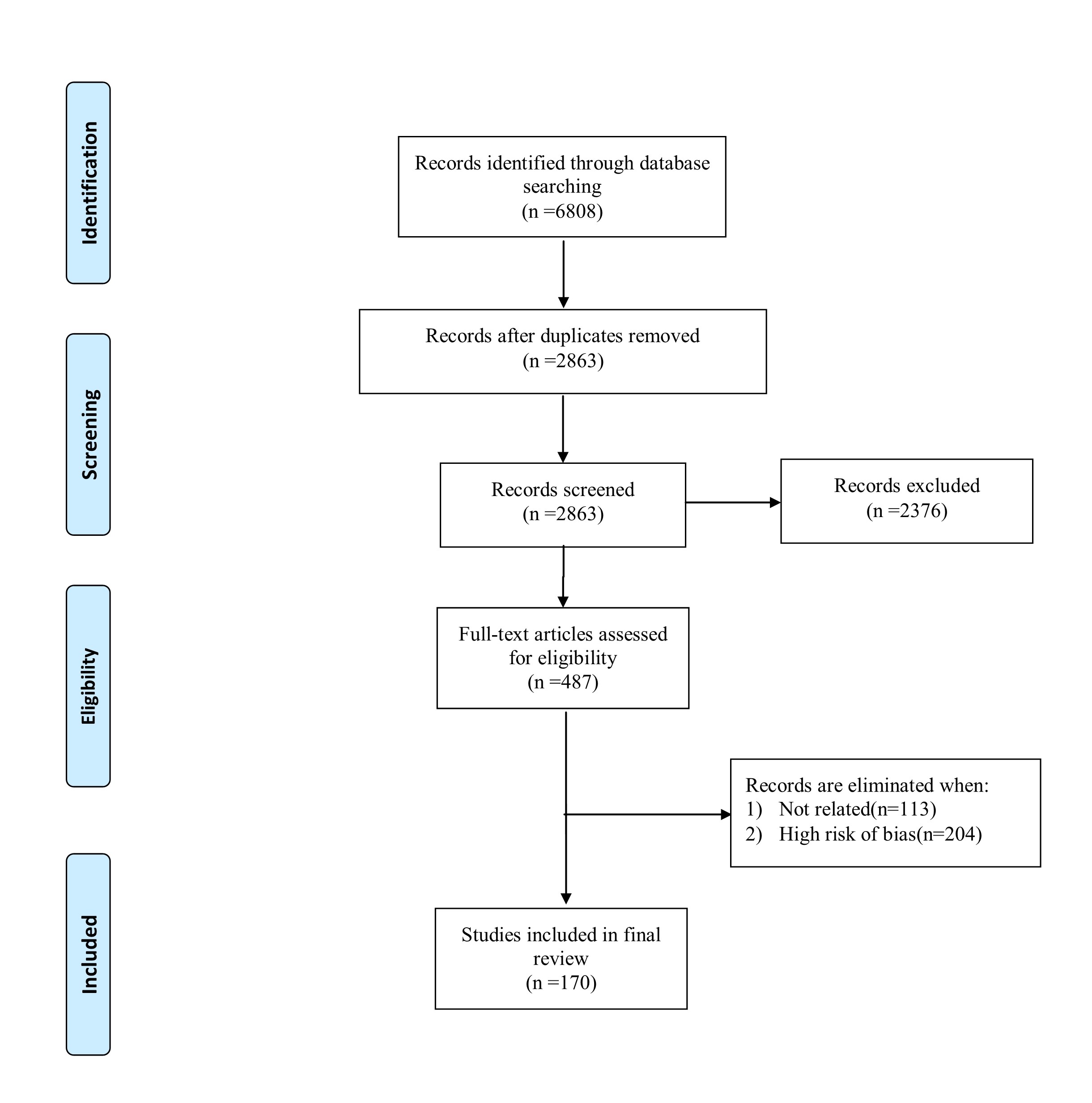

Supplement: Supplementary file 1 — SI-Figure 1 [file 41368_2021_125_MOESM1_ESM.jpg]
